# Preparation of Polyoxymethylene/Exfoliated Molybdenum Disulfide Nanocomposite through Solid-State Shear Milling

**DOI:** 10.3390/polym16101334

**Published:** 2024-05-09

**Authors:** Shuo Feng, Xinwen Zhou, Sen Yang, Jiayu Tan, Meiqiong Chen, Yinghong Chen, Huarong Zhang, Xu Zhu, Shulong Wu, Haidong Gu

**Affiliations:** 1 The State Key Laboratory of Polymer Material Engineering, Polymer Research Institute, Sichuan University, Chengdu 610065, China; 2 Baosheng Technology Innovation Corporation Limited, Yangzhou 225800, China

**Keywords:** molybdenum disulfide, polyoxymethylene, solid-state shear milling, exfoliation, dispersion

## Abstract

In this paper, the solid-state shear milling (S^3^M) strategy featuring a very strong three-dimensional shear stress field was adopted to prepare the high-performance polyoxymethylene (POM)/molybdenum disulfide (MoS_2_) functional nanocomposite. The transmission electron microscope and Raman measurement results confirmed that the bulk MoS_2_ particle was successfully exfoliated into few-layer MoS_2_ nanoplatelets by the above simple S^3^M physical method. The polarized optical microscope (PLM) observation indicated the pan-milled nanoscale MoS_2_ particles presented a better dispersion performance in the POM matrix. The results of the tribological test indicated that the incorporation of MoS_2_ could substantially improve the wear resistance performance of POM. Moreover, the pan-milled exfoliated MoS_2_ nanosheets could further substantially decrease the friction coefficient of POM. Scanning electron microscope observations on the worn scar revealed the tribological mechanism of the POM/MoS_2_ nanocomposite prepared by solid-state shear milling. The tensile test results showed that the pan-milled POM/MoS_2_ nanocomposite has much higher elongation at break than the conventionally melt-compounded material. The solid-state shear milling strategy shows a promising prospect in the preparation of functional nanocomposite with excellent comprehensive performance at a large scale.

## 1. Introduction

The two-dimensional molybdenum disulfide (MoS_2_) has attracted extensive attention due to its excellent tribological performance and potential electrical and optical performance [1]. The weak layered structure of MoS_2_ gives it its perfect lubrication property [2], which makes the MoS_2_ particles widely used as additives in lubricating oils, greases, and solid materials [3]. However, the morphology of the MoS_2_ particle has a great influence on its ultimate tribological performance. It was found that the exfoliated nanoscale MoS_2_ (such as fullerene-like, tube-like, and platelet-like) showed better tribological performance than the conventional bulk MoS_2_ [4,5]. Hu et al. [6] investigated the tribological properties of liquid paraffin filled with nano-ball MoS_2_, nano-slice MoS_2_, and bulk MoS_2_, respectively. The results showed that nano-ball MoS_2_ showed the better tribological performance, and nano-slice MoS_2_ performed the best lubrication property at high rotation speed in the hydrodynamic lubrication region. In recent years, the ultrathin nanoscale MoS_2_ has been reported to fabricate some field effect transistors due to its thickness-dependent bandgap [7] and is also used as a catalyst for the hydrogen evolution reaction [8]. As a result, the nano-MoS_2_ substance has been considered a promising candidate for various future electric devices. As a consequence, the preparation of nano-MoS_2_ has attracted increasing attention [9].

The bulk MoS_2_ is mainly composed of Mo-S-Mo sandwich layers, where every molybdenum sheet is interacted between two sulfur sheets. It was found that there are three types of crystalline MoS_2_, including 1T, 2H, and 3R [10]. Among them, 2H-MoS_2_ appears to have the most outstanding tribological performance. The Mo and S atoms are connected by a chemical bond within the layer, and the neighboring layers are weakly stacked and connected through Van der Waals forces [11]. Thus, the bulk MoS_2_ can be exfoliated to a few-layer MoS_2_ under appropriate conditions. In recent years, micromechanical cleavage [12] and various chemical exfoliation methods [13] (such as hydrothermal synthesis, vapor phase decomposition, and laser thinning) have been reported to prepare ultrathin MoS_2_. The micromechanical cleavage showed high quality and simplicity, but was limited to difficult production at a large scale. On the other hand, the chemical exfoliation method presented a relatively complicated procedure. Therefore, a simple and effective method for large-scale fabrication of nano-MoS_2_ with low cost has not been reported thus far. It is also reported that the previously prepared nano-MoS_2_ particles had a high surface energy and tend to be agglomerated when melt-compounded with the polymer matrix directly [14]. Xu [2] et al. confirmed that the MoS_2_ nanoplatelet particles presented bad dispersibility in rapeseed oil, which would lead to an increase in wear. Clearly, it is really extremely difficult to prepare the exfoliated nano-MoS_2_ with a good dispersion in the polymer matrix by only using an ordinary physical approach. This is because the interlayer spacing of the MoS_2_ is extremely small (as low as 0.65 nm).

Solid-state shear milling (S^3^M), a technology based on our self-designed pan-milling equipment [15], has been well developed in our laboratory for many years. This technology has been successfully adopted to achieve the preparation of highly filled polymer composites [16], ultrafine grinding of polymer/inorganic micro-nano composites [17,18], and recycling of wasted polymers [19,20]. The oppositely inlaid twin mill-pans of the above-mentioned pan-milling equipment can act as three-dimensional scissors during pan-milling, which could exert very strong compression, shear, and hoop stretching stresses for pulverization, dispersion, mixing, and mechanochemical activation on the milled materials. In our team’s previous work, the S^3^M strategy was able to efficiently cut the multi-wall carbon nanotubes and induce strong interfacial interactions with the polyamide 6 matrix [21]. This technology proved to effectively solve the compatibility problem and control the phase morphology of the nano-fillers in the polymer matrix. On this basis, the S^3^M technology is expected to be a potential and new simple physical approach to exfoliate and disperse MoS_2_ in a polymer matrix in a solid state. The polymer polyoxymethylene (POM) is an engineering plastic with excellent processability, mechanical, and tribological performance. It had been widely used as a self-lubrication material in many fields, such as engineering, automotive, and aerospace [22]. However, the neat POM still cannot satisfy the requirements as a sliding part in some extreme conditions, especially for those applied in ultra-small mechanical sliding devices with high temperatures and high loads [23]. Therefore, compounding with nano-MoS_2_ particles seems like a feasible method to further improve tribological performance and expand the application fields of POM.

Accordingly, in this work, the S^3^M technology was used to mechanochemically treat the mixture of pristine bulk MoS_2_ and POM at a solid state and on a large scale, aiming to effectively exfoliate and disperse the MoS_2_ particles in the POM matrix without aggregation, taking advantage of the very strong pulverization, dispersion, mixing, and mechanochemical activation functions. For comparison, the conventional POM/MoS_2_ composite was also prepared by twin-screw melt-compounding extrusion. The morphology and dispersion of MoS_2_ in the POM matrix, as well as the mechanical and tribological properties of the prepared POM/MoS_2_ nanocomposite, were fully investigated. This S^3^M strategy can successfully and efficiently realize the nano-exfoliation of MoS_2_ particles at a large scale, and the obtained POM/MoS_2_ nanocomposite exhibits excellent tribological and mechanical performance, thus exhibiting promising application prospects.

## 2. Experimental

### 2.1. Material

POM (M90), with an 80,000–100,000 average molecular weight, melt flow index of 9 g/10 min at 190 °C, and density of 1.4 g/cm^3^, was purchased from Yuntianhua Group, Chongqing, China. MoS_2_ with a particle size less than 47 μm was purchased from Tianjin Chemical Industry, Tianjin, China.

### 2.2. Sample Preparation

#### 2.2.1. Preparation of POM/MoS_2_ Composite

The MoS_2_ was first mixed with POM pellets in a high-speed mixer, and the loading of MoS_2_ was fixed at 15 wt%. Then, the pan-milling equipment was applied to pulverize and mill the mixture with a rotation speed of 20 rpm. The discharged co-powders were collected for the next milling cycle. The heat generated during pan-milling was removed by the circulating water. A small quantity of the milled POM/MoS_2_ co-powders was collected for characterization every 10 milling cycles. After 40 milling cycles were finished, the obtained POM/MoS_2_ co-powders were firstly dried in an oven at 60 °C for 12 h and then used as the master batch and diluted to the MoS_2_ content at 2 wt% by adding the dried neat POM and 0.5 wt% of the antioxide agent IRGANOX 245 to avoid mechanochemical degradation. Subsequently, the well-mixed mixtures with 2 wt% MoS_2_ were extruded in a twin-screw extruder (Φ = 25 mm, L/D = 33, Chenguang Research Institute of Chemical Industry, China) with a screw rotation speed of 120 rpm at 180 °C, and the cooled extrudates were cut into pellets and dried. For purposes of comparison, the pristine MoS_2_ particles were also well mixed with the dried neat POM and 0.5 wt% of the antioxide agent IRGANOX 245 in a high-speed mixer and then simply melt-compounded in the above-mentioned twin-screw extruder under the same conditions.

#### 2.2.2. Preparation of POM/MoS_2_ Composite Samples for Tensile and Tribological Tests

The dumbbell-shaped specimens with a dimension of 150 mm × 20 mm × 4 mm (L × W × T) for the tensile test were prepared by using a MA500II injection molding machine (Ningbo Haitian Co., Ltd., Ningbo, China) with an injection speed of 50 mm/s at 180 °C. The samples for the tribological test were first compression-molded into sheets at 180 °C with 20 MPa and then cut into samples with a dimension of 30 mm × 7 mm × 6 mm (L × W × T).

### 2.3. Characterization

**Figure 1:**
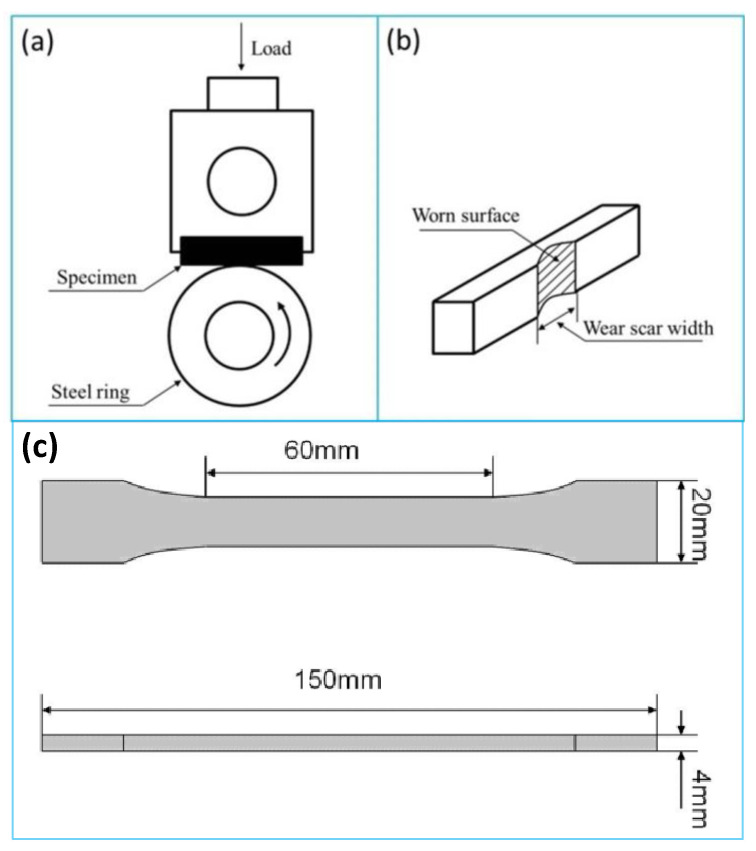
The schematic diagram of wear testing experiment for wear tester (**a**) and worn surface of sample (**b**); the shape and dimensions of tensile specimen (**c**).

The scanning electron microscope (SEM) morphology of the S^3^M (milled) and conventionally melt-compounded (unmilled) POM/MoS_2_ composites was observed on an Inspect F Scanning electron microscope (FEI Company, Hillsboro, OR, USA) with an accelerating voltage of 20 kV. Before observation, the worn scar of samples after the friction and wear tests and also the fractured surface of samples after tensile tests were coated with a thin gold layer to prevent charging on the surface. The polarized light microscope (PLM) morphology was observed by a DM2500p microscope (Leica Camera AG, Wetzlar, Germany). Before observation, the samples were meticulously prepared by cryogenically ultramicrocutting them into 20 μm slices from the injection-molded sheet using an ultramicrotome machine. The milled and unmilled POM/MoS_2_ composites were melted at 180 °C and controlled by the THMSG600 heating and cooling stage (Linkam Scientific Instruments, Salfords, UK). The X-ray diffraction (XRD) analysis of the milled and unmilled POM/MoS_2_ composites was performed using a DX-1000 diffractometer (Dandong Fangyuan Instrument Co., Ltd., Dandong, China). The CuKα generator system was operated at 40 kV and 25 mA, and the scanning 2θ ranged from 5° to 35°. The transmission electron microscope (TEM) was performed on a Tecnai G2 F20 electron microscope (FEI Company, Hillsboro, OR, USA) with an accelerating voltage of 200 kV. The injection-molded samples of milled and unmilled POM/MoS_2_ composites were cryogenically ultramicrocut into 80–100 nm thin slices at −100 °C using a LEICA EM FC6 frozen ultramicrotome. The POM/MoS_2_ thin films were then placed on the copper grids for observation. The Raman measurements were conducted on the pristine MoS_2_ and POM/MoS_2_ co-powders with 40 milling cycles by using a LabRAM HR Laser Raman spectrometer (HORIBA Company, Palaiseau, France) at room temperature with an excitation wavelength of 532 nm. The friction and wear tests were conducted using a MC-200 friction and abrasion testing machine (Beijing Guance Testing Instrument Co., LTD., Beijing, China) with a block-on-ring arrangement at room temperature with a rotation of 120 rpm and a load of 200 N for 60 min. The schematic diagram of the friction and wear testing experiment is shown in Figure 1a. The wear loss was determined by the wear scar width (Figure 1b). The friction torques (T) were recorded every second, and the friction coefficient (μ) was defined by an equation of μ = T/MR, where μ was the friction coefficient, T was the average friction torque (Nm), M was the load (N), and R was the radius of the steel ring (m), respectively.

The tensile property measurement of the milled and unmilled POM/MoS_2_ composites was performed on injection-molded dumbbell-shaped specimens using a 5567 type of Instron Universal Testing machine (Instron Company, Buckinghamshire, UK) with a cross-head rate of 10 mm/min at room temperature.

## 3. Results and Discussion

### 3.1. Preparation and Structure Characterization of POM/MoS_2_ Nanocomposite

#### 3.1.1. Morphology Evolution of S^3^M POM/MoS_2_ Co-Powders

**Figure 2:**
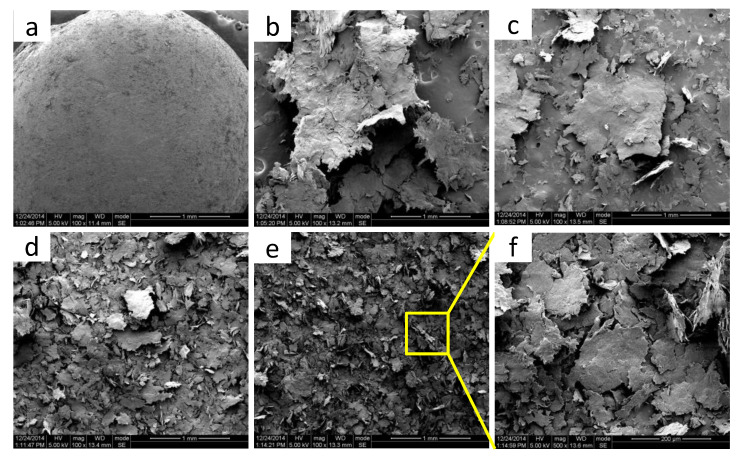
The SEM images of POM/MoS_2_ co-powders prepared at different milling cycles: 0 cycles (**a**); 10 cycles (**b**); 20 cycles (**c**); 30 cycles (**d**); 40 cycles (**e**); magnified image with 40 milling cycles (**f**).

The morphology development of POM/MoS_2_ co-powders prepared at different milling cycles was observed by SEM, and the results are shown in Figure 2. As can be seen, the MoS_2_ particles are adhered to the ball-like POM particles through simple physical mixing (in a high-speed mixer) before pan-milling treatment. The milled POM/MoS_2_ co-powder particles with low milling cycles (10) show a sheet structure under the effect of the very strong compression, shear, and hoop stretching stress fields generated by pan-milling. It is noted that the sheet-like POM powder particles possess a relatively higher specific surface area and benefit from sufficient contact with MoS_2_ particles. Moreover, the MoS_2_ particles have the opportunity to be imbedded into the POM matrix under strong compression stress. As a consequence, it is promising to achieve good dispersion of MoS_2_ filler particles in the POM matrix by using this strategy. With increasing milling cycles, the POM/MoS_2_ co-powders keep the same sheet structure, but the size of the sheet particles becomes much smaller and thinner, indicating the high efficiency of the S^3^M technology. The final size of the POM/MoS_2_ co-powder particles with 40 milling cycles could further decrease to 200 μm with the effect of the continuous pulverization and mixing provided by S^3^M.

#### 3.1.2. The Dispersion of MoS_2_ Particles in POM Matrix

**Figure 3:**
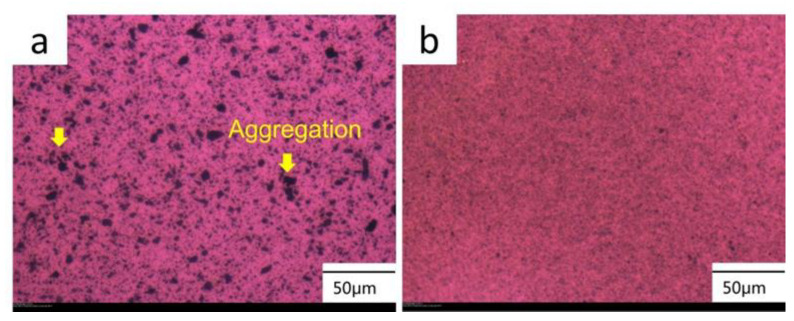
The PLM images of POM/MoS_2_ composite prepared by different method at 180 °C: conventionally melt-compounding (**a**) and S^3^M method (**b**).

The dispersion behavior of the incorporated MoS_2_ particles in the conventionally unmilled and milled POM/MoS_2_ composite was observed using PLM. The results are shown in Figure 3. Obviously, for the conventionally melt-compounded sample (Figure 3a), the opaque black areas represent the MoS_2_ particles, which basically appear to have a size in the range of microns. There is a heavy agglomeration of MoS_2_ particles occurring in the POM matrix, demonstrating the poor dispersion effect of twin-screw extrusion processing. This could be due to the high surface energy and interaction potential of the MoS_2_ particles. Comparatively, for the pan-milled sample (Figure 3b), the dispersion of MoS_2_ particles in the POM matrix is substantially improved. The individual MoS_2_ particle cannot be clearly identified due to the great reduction in filler particle size after milling. The above results again verify the high efficiency of S^3^M technology. This is because the MoS_2_ particles are efficiently pulverized and imbedded into the POM matrix under the strong compression, shear, and hoop stretching stresses induced by pan-milling. Thus, the S^3^M strategy could be applied to effectively solve the aggregation problem of the pristine bulk MoS_2_ particles in the POM matrix.

#### 3.1.3. Crystal Structure of S^3^M POM/MoS_2_ Co-Powders

**Figure 4:**
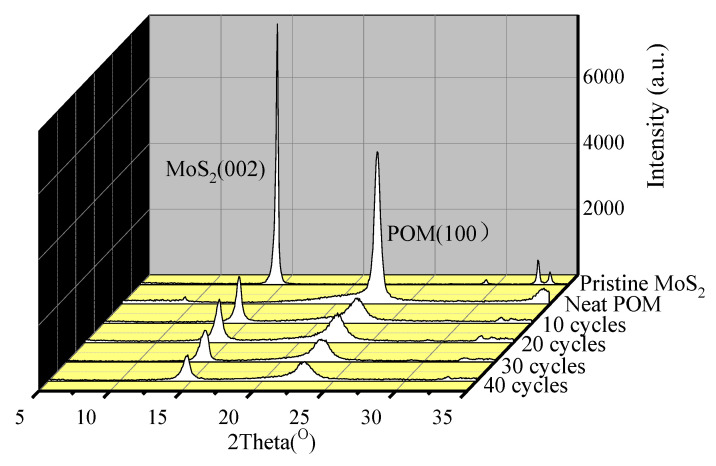
The XRD patterns of pristine MoS_2_, neat POM and POM/MoS_2_ co-powders prepared at different milling cycles.

Figure 4 shows the XRD patterns of pristine MoS_2_, neat POM, and POM/MoS_2_ co-powders with different milling cycles. As can be seen, the diffraction patterns of MoS_2_ mainly appear in three peaks at 14.6°, 32.8°, and 33.7° in the detected 2θ range, which correspond to the (002), (100), and (101) crystal planes of 2H-MoS_2_ [24], respectively. The diffraction peaks located at 22.8° and 34.6° are attributed to the (100) and (105) crystal plane of POM, respectively [25]. As can be seen, the diffraction peak intensities of MoS_2_ and POM decrease sharply after 10 milling cycles, indicating the breakage of original crystal crystallites and distortion of the three-dimensional crystalline order of MoS_2_ after the S^3^M process [26]. In addition, the diffraction peak intensities of MoS_2_ (002) and POM (100) show a decreasing tendency with further increase of milling cycles. According to the Scherrer equation:

(1) $$D=\frac{k\lambda }{\beta \cos\theta }$$

where *D* is the crystalline size (nm), *λ* is the X-ray wavelength in nanometer (nm), *β* is the full width at half maximum (FWHM), *k* is the scherrer constant, and *θ* is the diffraction angle.

Accordingly, the crystalline size can be calculated by using the full width at half maximum (FWHM) of the diffraction peak and diffraction angle. Apparently, the crystalline size of MoS_2_ decreases with increasing milling cycles, indicating the constant milling treatment could destroy the inner-ordered stacking of MoS_2_. Hence, the layered structures of MoS_2_ are expected to be exfoliated using the S^3^M strategy, which will be further confirmed by the following TEM characterization. Moreover, the diffraction profiles of MoS_2_ particles with different milling cycles show similar featured peaks, implying that the 2H crystal type of MoS_2_ does not change after pan-milling.

#### 3.1.4. The Microscopic Morphology of MoS_2_ in the POM Matrix

**Figure 5:**
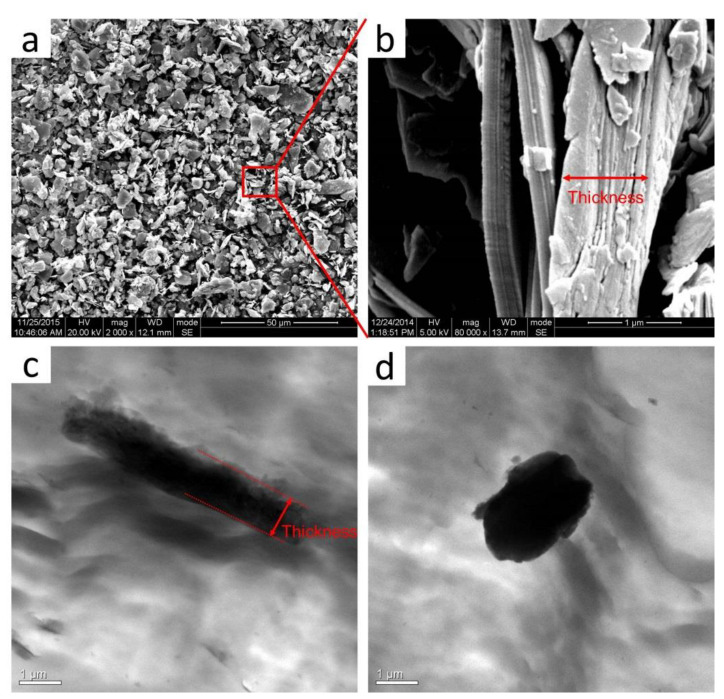
The SEM images of pristine MoS_2_ (**a**,**b**); The TEM images of the conventionally melt-compounded POM/MoS_2_ composite (**c**,**d**).

The dispersion morphology and crystallization structure of pan-milled POM/MoS_2_ nanocomposite were well explored, but the ultimate microscopic morphology of MoS_2_ particles after S^3^M processing is still not clear. Figure 5a,b show the SEM images of the pristine bulk MoS_2_ particles. As can be seen, most of the pristine MoS_2_ particles show a size distribution in the micron range, and some particles have a length dimension up to 20 μm. Meanwhile, the stacking layered structure can be clearly observed (Figure 5b, enlarged image), and the thickness of some large-size particles could achieve 1 μm. The morphology of MoS_2_ in the conventionally unmilled composite is observed by TEM (Figure 5c,d), and the dark area indicates the MoS_2_ particles. As can be seen, the thickness stays at the micron scale. MoS_2_ exists as pristine bulk agglomerates in the POM matrix, and the poor shear stress field of the twin-screw extruder could not induce the structural change of the pristine MoS_2_ particles.

**Figure 6:**
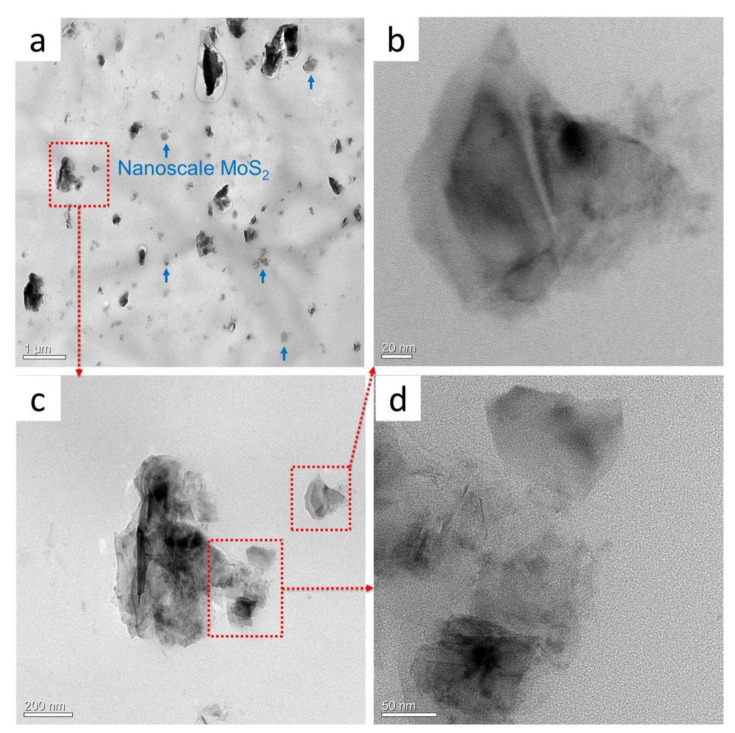
The TEM image of S^3^M-processed MoS_2_/POM nanocomposite (**a**), and the local magnifications of the individual exfoliated MoS_2_ particles (**b**–**d**); (**c**) towards (**a**), and (**b**,**d**) towards (**c**).

As a comparison, the morphology of MoS_2_ particles in the milled POM/MoS_2_ nanocomposite was observed using TEM, and the results are shown in Figure 6. As can be seen from Figure 6a, a small number of larger-size MoS_2_ particles can still be observed, while the size is much smaller than that of the original pristine particles. Meanwhile, a large quantity of nanoscale particles with a dimension of about hundreds of nanometers can be clearly identified. Figure 6b–d show the magnified morphologies of these nanoscale particles. As can be seen, there are few-layer nanosheets of MoS_2_ particles formed in the polymer matrix, clearly indicating the successful exfoliation of bulk MoS_2_ particles taking advantage of the very strong three-dimensional shear stress field of pan-milling. This is a breakthrough in the preparation of the exfoliated MoS_2_ in its solid state by only adopting a simple physical strategy. Some details could be further known from Figure 6b–d. For Figure 6b, the center region of particles appears translucent, implying only a few layers overlapping one another. The particle edge is transparent, indicating a much thinner structure in this area [8]. Figure 6c shows the TEM image of a larger particle (in small magnification). It can be seen that the center area appears opaque, while the edge region is translucent, indicating that these particles are partially exfoliated. Figure 6d further shows the magnified edge region of the larger particle, and it appears nearly transparent, apparently with a high exfoliation occurring here. It is noted that this region is weakly combined with the larger particle and seems to be separated from the larger one. Therefore, it can be speculated that these exfoliated nano-MoS_2_ were crushed and fractured on the surface of larger particles and finally delaminated from them via a S^3^M process.

#### 3.1.5. Raman Spectra of Pristine MoS_2_ and S^3^M MoS_2_/POM Nanocomposite

**Figure 7:**
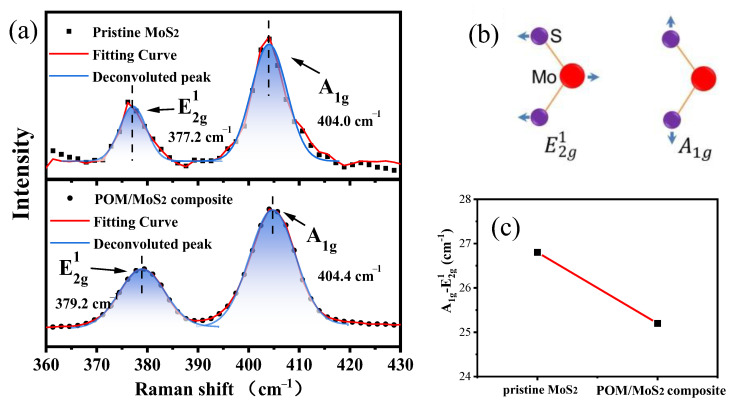
The Raman spectra of the pristine MoS_2_ and POM/MoS_2_ nanocomposite after 40 milling cycles (**a**), atomic displacement of the $E_{2g}^{1}$ and A_1g_ Raman active model (**b**) and frequency difference between the A_1g_ and $E_{2g}^{1}$ Raman modes (**c**).

In this section, Raman spectroscopy, which is widely applied to investigate the two-dimensional material for thickness identification, was used to further evaluate the exfoliation of MoS_2_ particles after pan-milling. Figure 7 shows the Raman spectra of pristine MoS_2_ and milled POM/MoS_2_ nanocomposite with 40 milling cycles. It can be seen that there are two main peaks clearly presented, which are corresponding to $E_{2g}^{1}$ (377.2 cm^−1^, in-plane vibration of two S atoms with respect to Mo atom) and A_1g_ (404.0 cm^−1^, out-of-plane vibration of S atoms) [27] (as shown in Figure 7b). Here, it is noted that the frequency of $E_{2g}^{1}$ increases after 40 milling cycles. It has been proven that the $E_{2g}^{1}$ frequency shifts in a high wavenumber direction due to the exfoliation of layered structure, which can be attributed to the influence of neighboring layers on the effective restoring force on atoms and the increase in dielectric screening of long-range Coulomb interactions [28]. As a consequence, the frequency difference between the A_1g_ and $E_{2g}^{1}$ decreases from 26.8 cm^−1^ to 25.2 cm^−1^ after S^3^M treatment, further verifying that the bulk MoS_2_ particles are successfully exfoliated into few-layer nanosheets of MoS_2_ [29].

### 3.2. Tribological Performance of POM/MoS_2_ Nanocomposite

**Figure 8:**
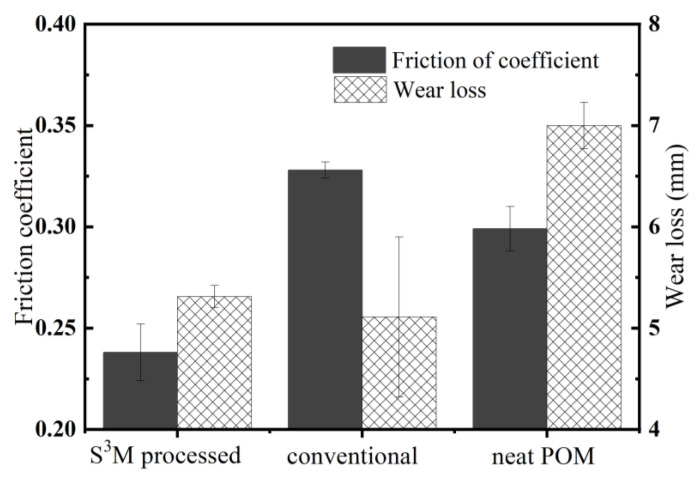
The friction coefficient and wear loss of milled composite, conventionally unmilled composite and neat POM.

The friction coefficient and wear loss of the milled nanocomposite, conventionally unmilled composite, and neat POM are shown in Figure 8. As can be seen, the pan-milled POM/MoS_2_ nanocomposite shows the lowest friction coefficient, while the conventionally melt-compounded POM/MoS_2_ composite presents an increase in friction coefficient when compared with neat POM, indicating that the exfoliated nano-MoS_2_ particles could really remarkably decrease the friction coefficient, while the pristine bulk MoS_2_ particles have the most negative influence on the friction coefficient. On the other hand, the S^3^M-processed and conventionally prepared composites present a lower wear loss than the neat POM, indicating the incorporation of MoS_2_ could effectively improve the abrasion property of POM.

**Figure 9:**
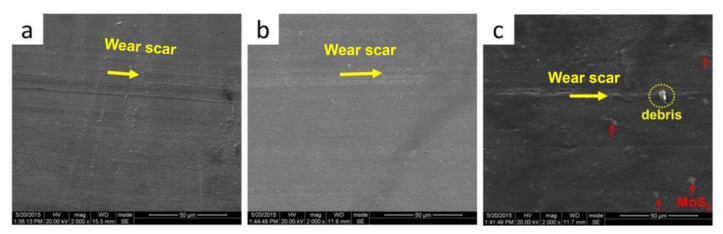
The SEM images of worn surface: neat POM (**a**), S^3^M-processed POM/MoS_2_ nanocomposite (**b**) and conventionally melt-compounded POM/MoS_2_ composite (**c**). The yellow arrow indicates the wear scar.

In order to deeply understand the effect of incorporated MoS_2_ on the friction and wear behaviors of POM, the morphology of the worn surface of neat POM, milled, and conventionally unmilled composites was investigated, and the results are shown in Figure 9. As can be seen, the worn surface of the neat POM appears to have obvious scratch grooves and some small debris. Many investigations [30,31] indicated that the wear mechanism of neat POM is governed by adhesion wear. Hence, the worn surface is plowed by its hard counterpart (spinning steel ring). Meanwhile, the plastic deformation may occur due to the lower hardness of the POM matrix. Additionally, the transfer film cannot be formed in neat POM [30], as a consequence of the higher wear loss of neat POM. The worn surface of S^3^M-processed POM/MoS_2_ nanocomposite appears the smoothest, and there are only some shallow scratch grooves, which can be observed. The improved wear resistance of the milled POM/MoS_2_ nanocomposite can be attributed to the formation of a 2H-MoS_2_ transfer film on the counterpart surface [32]. The worn surface of conventionally melt-compounded POM/MoS_2_ composite appears roughest, and the obvious plow-like gaps can be identified clearly. This large-size debris in the scratch grooves could probably be caused by the big MoS_2_ agglomerates plowed out in the POM matrix during the test. Additionally, there are also large quantities of MoS_2_ particles observed on the worn surface. This confirms that the bulk 2H-MoS_2_ particles easily form the transfer film, and sliding then occurs on the MoS_2_ lubrication film, which can possibly explain the reason why the conventionally prepared POM/MoS_2_ composite could present a relatively lower wear loss.

**Figure 10:**
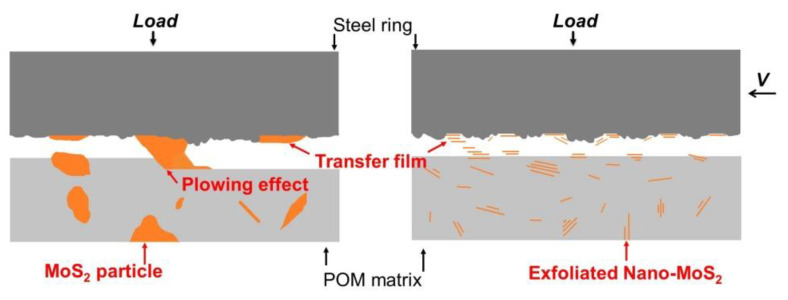
The schematic diagram of sliding process for the conventionally melt-compounded POM/MoS_2_ composite and S^3^M-processed POM/MoS_2_ composite.

Based on the above results, a friction and wear mechanism could be proposed. Figure 10 demonstrates the sliding process of the POM/MoS_2_ composite. In the conventionally prepared POM/MoS_2_ composite, there are large-size MoS_2_ particles (in the range of 1~30 μm) dispersed in POM, which play a primary role in separating the frictional pairs. Besides, the two-dimensional MoS_2_ generally possesses an extremely high strength perpendicular to the thickness direction [33], which could probably plow out the POM in the sliding process, leading to the obvious scratch grooves. However, the pan-milled POM/MoS_2_ nanocomposite appears to have the smoothest worn surface, indicating that the exfoliated nano-MoS_2_ particles could improve the anti-wear performance. The size of the exfoliated nano-MoS_2_ particle (in the range of nanometers) is smaller than the surface roughness (0.8 μm) of the steel ring used. Thus, the ultrathin MoS_2_ particles can easily enter the contact area of the steel ring and then prevent the POM matrix from being worn [34]. The overall friction coefficient *μ* in a tribological test can be divided into two parts, i.e., the adhesion term *μ_a_* and the plowing term *μ_p_*. Certainly, the friction coefficient *μ* can be defined by the equation *μ* = *μ_a_* + *μ_p_* [35]. Obviously, the lowest friction coefficient of the milled POM/MoS_2_ nanocomposite can be attributed to the lower ratio of plowing due to the smoothest worn surface. Comparatively, the obvious scratches of the conventionally prepared POM/MoS_2_ composite mean a higher ratio of plowing term, certainly resulting in a higher coefficient. As a result, the S^3^M-processed POM/MoS_2_ nanocomposite can have much better tribological performance.

### 3.3. Mechanical Performance of POM/MoS_2_ Nanocomposite

**Figure 11:**
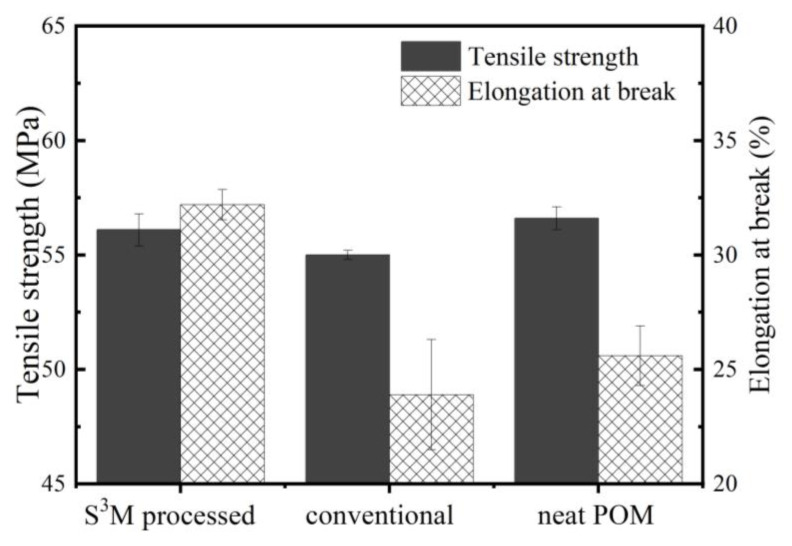
The mechanical property of S^3^M-processed POM/MoS_2_ composite, conventionally melt-compounded POM/MoS_2_ composite and neat POM.

Figure 11 shows the mechanical performance of different samples. It can be seen that the tensile strength of the milled and unmilled POM/MoS_2_ composites is slightly lower than that of neat POM, possibly due to the degradation effect of MoS_2_. Obviously, the slight decrease would have little influence on the ultimate application of S^3^M-processed POM/MoS_2_ nanocomposite. Compared with the conventionally prepared POM/MoS_2_ composite and neat POM, the elongation at break of the S^3^M-processed POM/MoS_2_ nanocomposite increases remarkably. Very clearly, the excellent comprehensive performance of POM/MoS_2_ nanocomposite can be obtained using S^3^M technology.

**Figure 12:**
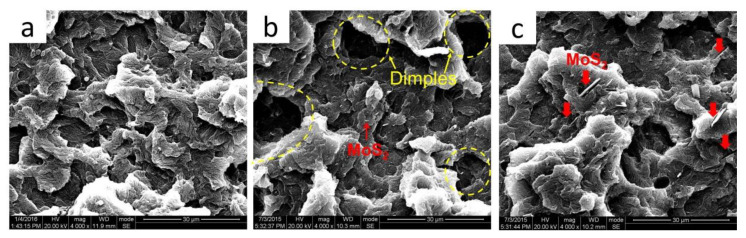
The SEM images of the fractured surface of different samples after tensile test: neat POM (**a**), S^3^M-processed POM/MoS_2_ nanocomposite (**b**) and conventionally melt-compounded POM/MoS_2_ composite (**c**).

In order to deeply understand the influence of the S^3^M process on the mechanical performance, the fractured surface of the sample after the tensile test was observed by SEM, and the results are shown in Figure 12. As can be seen, the fractured surface of S^3^M-processed POM/MoS_2_ nanocomposite is uneven, and there are some deep dimples there, which can absorb a substantial amount of energy during a tensile test. On the other hand, the dot-like MoS_2_ particles appear nearly invisible, possibly due to the nanoscale dispersion, and the extremely small, rigid MoS_2_ particles would lead to local crazes, which benefit yielding and plastic deformation under stress fields [36]. As a result, the S^3^M-processed nanocomposite can have higher elongation at break. The fractured surface of the unmilled POM/MoS_2_ composite also appears uneven, but the dimples have almost disappeared. There are some large MoS_2_ particles that can be identified, and there are also some holes caused by the pulling out of MoS_2_ particles upon fracture, indicating the bad compatibility between MoS_2_ and POM. In addition, these large MoS_2_ particles would lead to stress concentration. As a result, the conventionally prepared POM/MoS_2_ composite shows poor mechanical properties.

## 4. Conclusions

The solid-state shear milling (S^3^M) technology was adopted to prepare the POM/MoS_2_ nanocomposite. As a comparison, the conventional melt-compounding method was also performed to prepare the POM/MoS_2_ composite in a twin-screw extruder. The dispersion and exfoliation of MoS_2_ particles, tribological properties, and mechanical performance of the above-prepared POM/MoS_2_ composites were comparatively investigated. The results show that the S^3^M strategy has a much better dispersion and exfoliation effect on the MoS_2_ particles than the traditional melt-compounding method. Under the effect of the very strong three-dimensional shear stress field induced by S^3^M, the pristine bulk MoS_2_ particles were pulverized into nanoscale particles and particularly efficiently exfoliated to few-layer 2H-MoS_2_ nanosheets at a large scale, which is verified by TEM, Raman, and XRD measurements. The dispersion of MoS_2_ particles in the POM matrix has accordingly improved substantially. On the contrary, the simple melt-compounding extrusion does not have any influence on the dispersion and exfoliation of MoS_2_ particles, and there is heavy agglomeration of filler particles in the matrix due to the poor shear stress field of the twin-screw extruder. Correspondingly, the S^3^M-processed POM/MoS_2_ nanocomposite shows substantially better tribological and mechanical properties than the traditionally melt-compounded material. Although incorporation of MoS_2_ could improve the anti-wear performance of POM, the S^3^M-processed nanocomposite shows a significantly lower friction coefficient due to nanoscale MoS_2_ decreasing the plowing effect. Meanwhile, the successfully exfoliated MoS_2_ nanosheets of S^3^M could substantially enhance the elongation at break of the POM/MoS_2_ composite. Therefore, the S^3^M strategy could show a very promising prospect in the preparation of POM/MoS_2_ functional nanocomposites with excellent comprehensive performance.

## Data Availability

Data are contained within the article.
